# Correlation between Olive Oil Intake and Gut Microbiota in Colorectal Cancer Prevention

**DOI:** 10.3390/nu14183749

**Published:** 2022-09-10

**Authors:** Raffaella Memmola, Angelica Petrillo, Sara Di Lorenzo, Sara C. Altuna, Baker Shalal Habeeb, Alessio Soggiu, Luigi Bonizzi, Ornella Garrone, Michele Ghidini

**Affiliations:** 1 Department of Biochemical, Surgical and Dental Sciences, University of Milan, 20122 Milan, Italy; 2 Medical Oncology Unit, Ospedale del Mare, 80147 Naples, Italy; 3 School of Medicine, University of Study of Campania “L. Vanvitelli”, 80131 Naples, Italy; 4 Medical Oncology, Oncomedica C.A., Caracas 1060, Venezuela; 5 Oncology Department, Shaqlawa Teaching Hospital, Shaqlawa City 44005, Iraq; 6 Oncology Unit, Fondazione IRCCS Ca’ Granda Ospedale Maggiore Policlinico, 20131 Milan, Italy

**Keywords:** extra virgin olive oil, microbiota, colorectal cancer, olive oil, antioxidants, EVOO

## Abstract

Extra virgin olive oil (EVOO) is a mainstay of the Mediterranean diet with its excellent balance of fats and antioxidant bioactive compounds. Both the phenolic and lipid fractions of EVOO contain a variety of antioxidant and anticancer substances which might protect from the development of colorectal cancer (CRC). The function of the intestinal microbiome is essential for the integrity of the intestinal epithelium, being protective against pathogens and maintaining immunity. Indeed, dysbiosis of the microbiota alters the physiological functions of the organ, leading to the onset of different diseases including CRC. It is known that some factors, including diet, could deeply influence and modulate the colon microenvironment. Although coming from animal models, there is increasing evidence that a diet rich in EVOO is linked to a significant reduction in the diversity of gut microbiome (GM), causing a switch from predominant bacteria to a more protective group of bacteria. The potential beneficial effect of the EVOO compounds in the carcinogenesis of CRC is only partially known and further trials are needed in order to clarify this issue. With this narrative review, we aim at discussing the available evidence on the effect of olive oil consumption on GM in the prevention of CRC.

## 1. Introduction

The Mediterranean diet (MD) is an eating pattern typical of Mediterranean Sea countries and involves the consumption of cereals, pulses, fruit, vegetables, extra virgin olive oil (EVOO) and a moderate intake of animal products. This dietary style is responsible for lower incidences of chronic diseases, including obesity, cardiovascular, and gastrointestinal diseases. Indeed, individuals who follow a Western diet characterized by a high intake of saturated fats and refined sugars are at higher risk of developing cancer than those who eat MD [1,2].

The best fat source of MD is EVOO, a high-energy food composed mainly of lipids, with an excellent balance of monounsaturated (MUFA, e.g., oleic acid), polyunsaturated (PUFA, e.g., linolenic acid), and saturated (SFA, e.g., stearic acid) fats and other bioactive compounds such as polyphenols [1].

Some bioactive components of EVOO (e.g., hydroxytyrosol and oleuropein) induce cell cycle arrest and cell death in colon cancer cells; therefore, EVOO can be considered effective against colorectal cancer (CRC) [1]. The COLON study found out that individuals with CRC treated with chemotherapy have an altered balance of intestinal flora that can lead to gastrointestinal disorders such as diarrhea, nausea and vomiting. EVOO and its bioactive compounds have positive effects on the microbiota and improve intestinal integrity as they inhibit pathogens, stimulate the growth of beneficial microorganisms and have antioxidant activity [2].

The aim of this work is to provide an update on the effect of olive oil consumption on gut microbiota in the prevention of CRC.

## 2. Quality of Olive Oils on the Market

The term “olive oil” is mistakenly used to identify all oils resulting from the processing of olives, encompassing a range of products with different qualities and characteristics. In reality, oils are classified based on olive quality, percentage of free acidity (expressed as oleic acid), processing and organoleptic analysis into EVOO, virgin olive oil and lampante olive oil [3].

Free acidity percentage is an essential parameter for assessing the quality of the oil; the lower its free acidity, the higher its quality. Free acidity expresses the percentage of free fatty acids present into the oil. In case of increase of free acidity, a series of changes occur leading to the formation of compounds that worsen oil’s organoleptic characteristics. Virgin oils with low acidity come from healthy olives, harvested from the tree and processed immediately afterwards [3].

EVOO is qualitatively the best of olive oils; it must be free of defects and have a free acidity of less than 0.8 per cent.

In case of acidity inferior to 2%, oil will not be marketed as EVOO but as virgin olive oil. The latter differs from extra virgin not only in acidity but also in slight organoleptic defects [3].

Lampante olive oil is of lower quality, with more defects and >2% free acidity. It so called because in the past it was used as fuel for oil lamps. EVOO is considered to be a high-quality food due to its health properties derived from its excellent lipid composition and bioactive compounds [1,2,3].

## 3. Oil: Components and Features

Fats represent one of the three macronutrients that must be consumed in a healthy diet. They provide energy (9 cal/g) and are fundamental components of cell membranes. Moreover, they take part in the production of hormones and carry fat-soluble vitamins (vitamin A (retinol), vitamin D (cholecalciferol), vitamin E (tocopherol), vitamin K (phylloquinone)) [4].

Guidelines for a healthy diet suggest an amount of fat of 20–35 % of the daily calorie intake, with saturated fat less than 10%. The ratio of saturated to monounsaturated fats is crucial in the prevention of many diseases such as intestinal inflammation and cancer [3].

**Figure 1:**
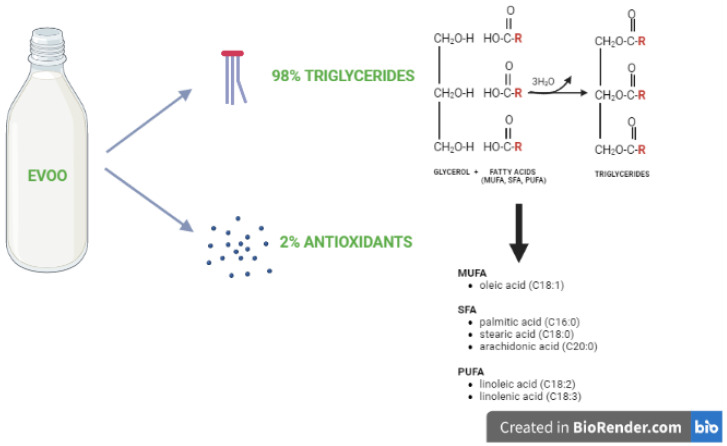
EVOO composition. EVOO consists of triglycerides formed from glycerol and fatty acids (monounsaturated [MUFA], saturated [SFA], and polyunsaturated [PUFA]) and antioxidants.

The fat of excellence in the MD is EVOO, consisting of approximately 98% triglycerides, made up of monounsaturated fatty acids (MUFA, 77%), such as oleic acid (C18:1); polyunsaturated fatty acids (PUFA, 8%), such as linolenic acid (C18:2) and linolenic acid (C18:3) and saturated fatty acids (SFA,15%), such as palmitic acid (C16:0), stearic acid (C18:0), and arachidonic acid (C20:0). The remaining 2% is composed of antioxidant micronutrients such as phenolic compounds (e.g., tocopherol, oleuropein and hydroxytyrosol), pigments (e.g., carotenoids and chlorophylls), sterols, and squalene [3] (Figure 1).

**Figure 2:**
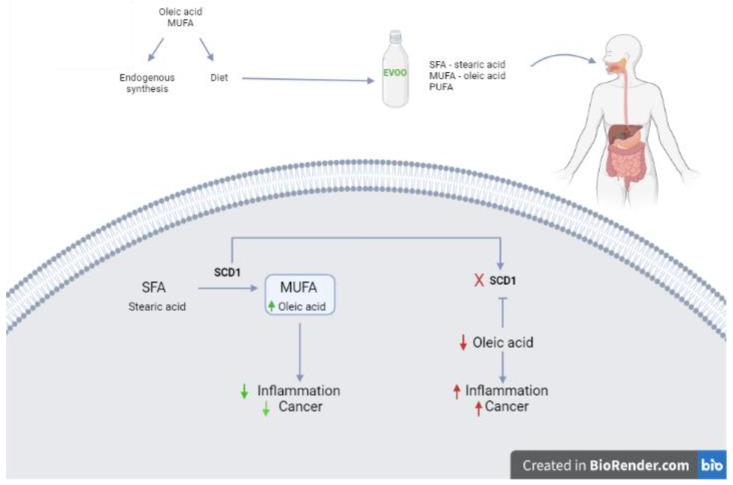
Regulation of oleic acid synthesis in humans. Oleic acid (MUFA) is synthesized endogenously by the enzyme Strearoyl-CoA Desaturase-1 (SCD1) and introduced with food such as EVOO. SCD1 catalyzes the conversion reaction of saturated fatty acids (SFA) to monounsaturated fatty acids (MUFA); if SCD1 is inhibited, the production of MUFA (Oleic acid) decreases. To prevent oleic acid deficiency, it is important to introduce it through the diet.

Oxidative stress is made of a set of alterations produced in cells exposed to an excess of oxidizing agents. Oxidants cause structural and functional alterations of their targets (membrane phospholipids, proteins and nucleic acids) leading to cell death. Oxidation is caused by oxygen free radicals (ROS) that damage fatty acids and cause lipid peroxidation resulting in loss of membrane function and integrity leading to apoptosis and necrosis [5]. The excellent balance of fats and the presence of antioxidant bioactive compounds in EVOO play a role in the prevention of different diseases such as cancer. However, oleic acid is a non-essential monounsaturated fatty acid insofar as the human body under physiological conditions is able to produce it endogenously. The enzyme that catalyzes this reaction is SCD1 (Strearoyl-CoA Desaturase-1), which is present in the intestinal epithelium. In particular, SCD1 is able to convert saturated fatty acids (SFA) into monounsaturated fatty acids (MUFA), promoting a reduction in inflammation and oxidative stress and preventing many diseases such as cancer [6] (Figure 2).

For example, stearic acid, a pro-inflammatory fatty acid, can be converted into oleic acid, a fatty acid with anti-inflammatory action. The inactivation of SCD1 causes increased intestinal inflammation and subsequent cancer promotion. Prevention of this process with a diet supplemented with oleic acid helps in restoring the normal intestinal physiology with reduced inflammation and suppression of tumor development [6].

## 4. Role of Olive Oil in Colorectal Cancer Prevention

The traditional MD is considered a well balance food schedule. This is also related to the presence of olive oil, whose antitumor properties are worth of interest especially in the Mediterranean area. Indeed, in this region the incidence of cancers is lower compared to other countries [7]. In particular, the beneficial effects of olive oil were reported in many kinds of tumors, including SKBR3 and MCF7 breast, A549 lung and HT115 colorectal cancer cell lines [3,8,9,10,11]. Indeed, a meta-analysis including data of 13,800 cancer patients and 23,340 controls from 19 studies showed that people with higher olive oil intake have a 34% lower chance to have any type of cancer [9].

There are several hypothesis and mechanisms that could be involved in cancer prevention process induced by olive oil. For instance, both the phenolic and lipid fractions of olive oil contain a variety of antioxidant and anticancer substances which might protect from the development of CRC. In this context, Gill et al. found that the phenol extract of EVOO prevents the in vitro cells attachment by hindering the binding of integrin to various types of extracellular matrix proteins (ECM) [12]. This study was the first to demonstrate the anti-invasive effects of phenols in olive oil. Taking into account that integrins are crucial for cell invasion and migration, fundamental steps in carcinogenesis initiation, promotion and subsequent metastatization, the findings of Gill et al. are relevant. Then, this study showed that the intake of oleic acid and PUFA induces apoptosis and cell differentiation mediated by an early downregulation of COX-2 followed by a reduction in the expression of Bcl-2.

It has been repeatedly highlighted how the constituents of EVOO interfere with DNA methylation and histone modifications, thus affecting the expression of genes involved in cancer development. In this context, a recent study by Bartoli et al. has shown how olive oil reduces the incidence of aberrant crypt foci in azoxymethane-treated rats [13].

Additionally, Di Francesco et al. showed that EVOO and its extracts act on the endocannabinoid system, which has been shown to be altered in cancer [14]. More in details, EVOO regulates the gene expression of the type 1 endocannabinoid receptor (CB1) through epigenetic mechanisms, such as inhibition of DNA methylation or histone acetylation. In human CRC cells (Caco-2 type) the CB1 gene is less expressed, leading to a DNA hypermethylation of its promoter Cnr1, and resulting in cell proliferation. These findings are in line with a previous study that showed higher rate of Cnr1 methylation in human CRC (77%) compared to hypermethylation of tumor suppressor and repair genes DNA, such as the cyclin-dependent kinase inhibitor P16/INK4a, P14/ARF, APC, hMLH1, and MGMT, which are methylated in a few cases (10–30%) [15]. Then, Di Francesco et al. evaluated the expression of four micro RNAs to further investigate the mechanisms at the basis of CB1 induction by EVOO. Among these, the expression of miR23a and miR-301 was selectively reduced after the administration of EVOO for one or ten days. Interestingly, these effects were mainly related to the phenol fraction of EVOO [14].

## 5. Gut Microbiota and CRC Development

The human being is made up of about 38 trillion bacteria of which about 1012 microbes make up the intestinal microbiota [16]. The function of the intestinal microbiome is essential for the integrity of the intestinal epithelium, being protective against pathogens and maintaining immunity. On the other hand, dysbiosis of the microbiota alters the physiological functions of the organ, leading to the onset of diseases [17]. Among them, CRC is one of the big killers and is a major global health burden. It ranks the third place in terms of incidence and the second in mortality worldwide [18].

Abnormalities in intestinal microbiota are referred to as “dysbiosis”, and are linked to inflammatory environments inside the intestinal mucosa, with a higher concentration of reactive oxygen species (ROS), a disproportion of B and T lymphocytes, and metabolism of short-chain fatty acids and other micronutrients that are directly linked to protection against metabolic and inflammatory diseases in the intestinal mucosa [19]. Inflammatory bowel disease is linked to a higher concentration of harmful bacteria, *Enterobacteriaceae*, *E. coli*, *Bacteroides* spp. and *Ruminococcus torques*, as well the growth of *Biophila wadsworthia* (which triggers cytotoxic Th1 response), due to the higher availability of SFA and low PUFA content present in vegetable oils, such as olive oil, among others. Eastern and MD, where these nutrients are more prevalent, tend to be linked to higher concentrations of *Prevotella* spp., also related to higher content of fiber, and lower ingestion of animal protein and fats, which stimulate growth of *Bacteroides* spp. [19,20]. The composition of the microbiome is directly linked to metabolic and inflammatory diseases, such as obesity, diabetes, allergies, and more recently, though more cautiously linked, a relevant role in CRC. In fact, the perpetuation of an inflammatory state helps stimulate the proliferation of cancer cells and the activation of proliferative pathways, such as NF-KB and β-catenin. Another hypothesis links several inflammatory bacteria present in the oral mucosa in patients with periodontal disease, since these pathogenic bacteria could enter the bloodstream via periodontal inflammation and reach the intestinal epithelium, where they contribute to an environment favorable for tumor formation [17,21].

Emerging studies have examined the correlation and the role of the gut microbiota (GM) in CRC carcinogenesis. These studies have shown how the composition of the microbiome, enriching or depleting certain microbes, can vary according to the state of health of the individual [17] (Table 1). For instance, *Streptococcus bovis*, a gram-positive coccus, is a reported risk factor for CRC [22,23], *Fusobacterium nucleatum* (*F. nucleatum*) is increased in human colorectal adenomas and carcinomas and may contribute to disease progression from adenoma to cancer, increasing especially in the early stages and showing a poor prognosis. Then, the commensal bacterium *Escherichia coli* (*E. coli*) is more represented in the mucous membrane of CRC patients than in those from healthy people [17]. Long et al. also found that the bacterium *Peptostreptococcus anaerobius* is significantly abundant in the feces and mucosa of CRC patients [24]. Conversely, the deficiency of certain microbes leads to the prevarication of those with harmful actions over the diners who have protective effects against CRC. As evidence of this, some bacteria, mostly probiotics, such as the butyrate producer *Clostridium butyricum* and lactate producer *S. thermophilus*, are absent in CRC patients [17]. Interestingly, due to the fact that the microbiome alterations could be detected in colorectal adenomas as well as in the early stage of CRC, it might be considered a sort of early biomarker in the CRC development process. Thus, taking into account the importance of GM in CRC prevention, today it is known that some factors, including diet, could deeply influence and modulate the colon microenvironment. More in details, some studies have shown how food, by modulating in part the composition and diversity of the intestinal microbiota, can impact on CRC at different stages of carcinogens, including promotion and metastatization. In particular, Zhang et al. showed that 38.3% of CRC cases were related to poor diets with low grains intake or rich in red and processed meats [25]. Additionally, in an unbalanced dietary pattern, microbial metabolism can generate pro-carcinogenic chemicals, such as secondary bile acids (in the high-fat diet) or N-nitroso compounds, and hydrogen sulfide regulatory T-cell differentiation (in high-protein diet).

## 6. Role of Olive Oil on Microbiota

Several interventions have been suggested in order to restore intestinal dysbiosis and lower risk of the diseases related to this phenomenon. The most extensively studied are dietary interventions including change in dietary patterns, lowering ingestion of animal proteins, refined sugars and SFA and increasing availability of PUFA, polyphenols, and dietary fibers, and an increase in physical exercise. Experimental analysis in animal models shows that increasing ingestion of vegetable fats, including olive oil, may be linked to an impact in GM, ultimately helping to change the predominant bacteria into a more protective group of bacteria, and help control and ultimately lower the development and progression of CRC [1,19]. Rodriguez-Garcia et al. described these findings in a recent study, where a high-fat diet was linked to a reduction in *Lactobacillus*, *Streptococcus*, *Turicibacter*, *Blautia*, *Clostridium*, *Ruminococcus*, and *Anaerostipes* and an increase in *Lactococcus*. In this experiment, the use of a diet high in EVOO was linked to a higher reduction of *Enterococcus* in a significant way. EVOO was also linked to an increase in the *Firmicutes/Bacteroidetes* ratio, lowering the presence of harmful bacteria and increasing the availability of favorable bacteria for the murine intestine. The mice that were fed a EVOO-high diet also showed a significant reduction in *Proteobacteria.* In general, a diet rich in EVOO was found to be linked to a significant reduction in the diversity of the GM in animal models. Although the study has several limitations, the findings show that a higher ingestion of vegetable fats, especially EVOO, is linked to a more favorable intestinal microbiota [19] (Table 1).

Although extrapolation from animal models to humans is difficult due to the different species of bacteria colonizing the intestinal mucosa, these findings in animal models are helpul. Indeed, they demonstrate the impact of dietary interventions on the development of disease, the response to treatment and the survival of patients, as well as the life expectancy in the general population [17]. More detailed studies in humans are needed to understand the relation between oral and intestinal flora and how dietary interventions can modify these factors. Some recent works included testing fecal samples with immunochemical tests to determine intestinal flora composition and proportion [26]. Grobbee et al. proposed a model of stable measurements of several bacteria including *Escherichia coli*, *Fusobacterium nucleatum* and *Bacteroidetes* in order to determine concentration and composition of intestinal flora. Results found that there is a higher bacterial load in patients with CRC and intestinal dysplasia. In this study, the tests carried out included isolation and amplification of bacterial DNA for the immunochemical tests. This can hinder availability for the implementation of this testing as a routine evaluation of patients. However, more studies on the composition of intestinal microbiota and changes in the intestinal microbiome due to dietary interventions will help clear the pathway to a better understanding of cancer biology and of overall evaluation of CRC patients [26].

## 7. Ongoing Clinical Trials

Despite the interest in this topic, the ongoing clinical trials in this field are few (Table 2). In this regard, the phase 3 NCT01048463 trial is evaluating whether Eicosapentaenoic Acid (EPA) in combination with enteral nutrition can improve nutritional/immunological status, quality of life and reduce chemotherapy-related side effects in patients receiving chemotherapy for patients affected by gastric and CRC. The use of EPA in these patients is based on preclinical evidences that EPA can modulate the immune system of the host and antagonize the metabolic and inflammatory changes induced by the tumor [27].

Then, NCT00475722 study is focusing on the development of CRC prevention diets by randomizing 120 people to eat according to a healthy diet or according to the MD. In particular, the MD differs from the first one due to an increase in *n*-3 and *n*-9 fatty acids in fruit and vegetable intake and a decrease in *n*-6 fatty acids [28].

Lastly, the NCT01070355 is a phase 2, double-blind, randomized, placebo-controlled study of EPA, which is evaluating the role of EPA in reducing tumor growth markers in patients with CRC and liver metastases who are candidates to receive a surgical approach. The trial has been completed and results are awaited [29].

## 8. Conclusions

There is emerging evidence on the correlation between dietary factors and GM in CRC prevention. Microbiota modulation is of key importance for CRC prevention. Different strategies, such as dietary interventions, probiotic use, and fecal microbiota transplantation have been employed for that purpose [17]. The consumption of dietary fibers, plant derived phytochemicals, probiotics, and EVOO revives the GM, while a low intake of fibers and excess intake of processed food and red meat lead to GM disturbances and increase the risk of CRC [30]. Although there are few studies evaluating the interaction between intestinal microbial composition and CRC occurrence, several findings support the role of olive oil polyphenols as onco-suppressor agents. Indeed, they are involved in different processes, such as inflammation, oxidative damage, and even epigenetic modulation. Despite increasing pre-clinical and clinical efforts, the evaluation and study of intestinal microbiota remains challenging due to individual variations, differences in tumor stages, and cross-species translation.

In conclusion, CRC is a multifactorial disease in which diet plays an important role. The traditional MD has long been thought of as a healthy lifestyle and olive oil is an important constituent of it. The potential beneficial effect of the EVOO compounds in the carcinogenesis of CRC is only partially known at the time of writing. Therefore, further trials are needed in order to clarify this issue and include EVOO in the list of CRC preventive agents.

## Figures and Tables

**Table 1:** CRC associated intestinal microorganisms and GM associated with CRC prevention [19,26].

Microorganisms Associated with CRC Increased Risk	Microorganisms Associated with CRC Prevention
*Streptococcus bovis* *Bacteroides fragilis* *Fusobacterium nucleatum* *Enterococcus faecalis* *Escherichia coli* *Peptostreptococcus anaerobius* *Staphylococcus* spp. *Pseudomonas* spp. *Neisseria* spp. *Sphingomonas* spp. *Prevotella* spp.	*Akkermansia muciniphila* *Bifidobacterium* spp. *Firmicutes* spp. *Lactococcus lactis*

Abbreviations: CRC: colorectal cancer; GM: gut microbiome; spp.: species plural.

**Table 2:** Main ongoing clinical trials exploring the role of olive oil compounds in the prevention and treatment of cancer patients.

Name of Study and NCT Number	Phase	Study Arms	N° of Patients	Study Type	Status
Phase 3 Study of Enteral Nutrition Rich in Eicosapentaenoic Acid (EPA) in Patients Receiving Chemotherapy for Gastric Cancer or Colorectal Cancer NCT01048463 [27]	Phase 3	Placebo Comparator: EN vs. Experimental: ENLDEPA (Nutriall+LDEPA+ Xelox) vs. Experimental: ENHDEPA (nutriall+HDEPA+ Xelox)	90	Interventional	Unknown status
A Mediterranean Diet in Colon Cancer Prevention NCT00475722 [28]	Not Applicable	Active Comparator: 1 Healthy Eating vs. Experimental: 2 Mediterranean	120	Interventional	Completed
The Effects of EPA on Biomarkers of Growth and Vascularity in Human Colorectal Cancer Liver Metastases (The EPA for Metastasis Trial) NCT01070355 [29]	Phase 2	Placebo Comparator: Placebo vs Active. Comparator: EPA free fatty acid	88	Interventional	Completed

Abbreviations: EN: enteral nutrition; EPA: Eicosapentaenoic Acid; vs.: versus; N°: number.
